# Two coronary tails, one tale: mirror-image distal origin anomalies

**DOI:** 10.1007/s10554-026-03696-8

**Published:** 2026-04-04

**Authors:** Scott P. David, Stefan Gherca, Michael J. Zellweger, Maurice Pradella, Philip Haaf

**Affiliations:** 1 Department of Radiology, Clinic of Radiology and Nuclear Medicine, University Hospital Basel, University of Basel, Basel, Switzerland; 2 Department of Cardiology, Cardiovascular Research Institute Basel, University Hospital Basel and University of Basel, Basel, Switzerland

Distal coronary origin anomalies with intercoronary continuation are exceedingly rare and represent a benign subset of coronary artery anomalies. They are most often detected incidentally during coronary CT angiography (coronary CTA) and their clinical relevance lies primarily in accurate anatomic recognition to ensure appropriate procedural planning [1]. We describe two patients with mirror-image distal anomalous origins of the right coronary artery (RCA) respectively circumflex coronary artery (CX) arising from the contralateral coronary artery.

Patient A (Panel A/B) underwent coronary CTA, which excluded high-grade obstructive coronary artery disease but revealed an anomalous coronary anatomy: The CX did not originate from the left main coronary artery (LMCA). Instead, a superdominant RCA system was present, with the CX arising from the distal RCA and coursing retrogradely within the left atrioventricular groove from caudal to cranial.

Patient B (Panel C/D) underwent CTA to exclude acute aortic dissection, which was ruled out but revealed a single LMCA with absence of an RCA origin from the right coronary sinus, consistent with a superdominant left coronary artery system. The RCA arose from the distal CX and followed a retrograde course within the right atrioventricular groove in a caudal-to-cranial direction.

**Figure:**
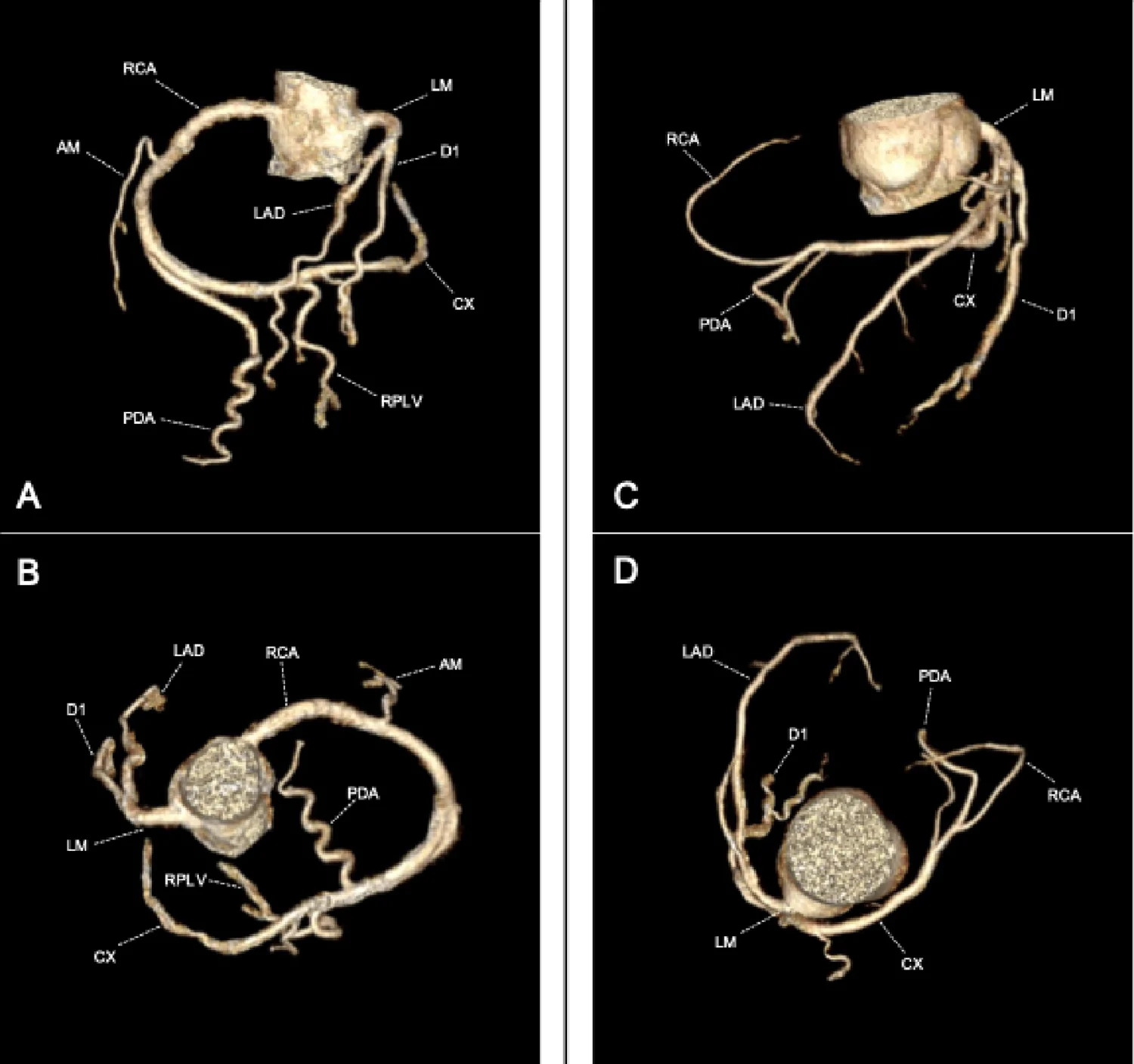

## Data Availability

No datasets were generated or analysed during the current study.
